# Age-Related Associations Between Relative Handgrip Strength and Body Composition in Women with Obesity

**DOI:** 10.3390/jcm15155938

**Published:** 2026-07-30

**Authors:** Luca Cavaggioni, Luisa Gilardini, Marina Croci, Silvia Martinelli, Paola Toja, Lucia Pasqualinotto, Simona Bertoli

**Affiliations:** 1 Obesity Unit and Laboratory of Nutrition and Obesity Research, Department of Endocrine and Metabolic Diseases, IRCCS Istituto Auxologico Italiano, 20145 Milan, Italy; l.gilardini@auxologico.it (L.G.); m.croci@auxologico.it (M.C.); s.martinelli@auxologico.it (S.M.); p.toja@auxologico.it (P.T.); l.pasqualinotto@auxologico.it (L.P.); simona.bertoli@unimi.it (S.B.); 2 Department of Theoretical and Applied Sciences, eCampus University, 22060 Novedrate, Italy; 3 Department of Food, Environmental and Nutritional Sciences (DeFENS), International Center for the Assessment of Nutritional Status (ICANS), University of Milan, 20133 Milan, Italy

**Keywords:** obesity, body composition, physical activity, correlation

## Abstract

**Background**: Relative muscular function has emerged as a clinically relevant marker of muscle quality and health in obesity. However, limited evidence is available regarding age-related associations between relative handgrip strength and body composition outcomes in women with obesity. **Methods**: This cross-sectional study included 419 female outpatients with obesity (age range 18–80 years). Participants were stratified into age groups according to sample distribution. Body composition was assessed using Body Mass Index (BMI), waist circumference (WC), fat mass percentage (FM%), fat-free mass (FFM%), and waist-to-height ratio (WHtR). Relative handgrip strength (relative HST) was assessed together with balance performance (M-BESS), lower-body functional performance (5STS), and movement quality (FMS CS). Kruskal–Wallis analyses were performed for all functional measures. Age-stratified linear regressions and pooled models including relative HST × age-group interaction terms were used to examine associations with body composition outcomes; whole-sample models were additionally adjusted for age and physical activity level (IPAQ-SF). **Results**: Significant differences across groups were observed for BMI (*p* = 0.0006), FM% (*p* = 0.0308), relative HST (*p* = 0.0002), M-BESS score (*p* < 0.0001), and 5STS performance (*p* < 0.0001). Relative HST was significantly associated with lower FM% and higher FFM% across all age groups. In addition, significant associations with lower WC were observed in middle-aged and older women. However, pooled interaction analyses provided no evidence that the associations between relative HST and FM%, FFM%, WC, or WHtR differed significantly across age groups (*p* of all interactions > 0.29). **Conclusions**: Relative HST was associated with body composition outcomes across different age groups of women with obesity, but the strength of these associations did not differ significantly by age group. These findings suggest that relative HST may provide clinically relevant information beyond traditional anthropometric measures. However, prospective studies are needed to determine its potential clinical utility in individualized, person-centered obesity management.

## 1. Introduction

Obesity is recognized as one of the most widespread chronic conditions worldwide and represents a major public health challenge, with estimates suggesting that approximately 1.12 billion individuals will be affected by 2030 [1]. Excess body weight accelerates age-related declines in physical function and represents a significant threat to independence and quality of life in older adults [2,3]. In addition to increasing the risk of chronic diseases such as diabetes and dyslipidemia, obesity is associated with altered locomotor biomechanics, impaired postural stability, and reduced physical function [4,5].

Muscular function and body composition are closely interconnected determinants of physical function and overall health in individuals with obesity [6,7]. Relative muscular function refers to muscular strength normalized to body weight and has emerged as a clinically relevant indicator of muscle quality and functional capacity in individuals with obesity [7]. Normalization to body weight was selected because it represents the most commonly adopted approach for assessing relative muscular function in individuals with obesity, allowing direct comparison with the existing literature.

Relative handgrip strength, the most widely adopted measure of relative muscular function, has been associated with body composition, cardiometabolic health, and functional outcomes in adult populations [6,8,9]. Motor competence, defined as the ability to efficiently perform a broad range of motor tasks and movement skills [10], represents a complementary indicator of physical function and is associated with functional capacity, participation in physical activity, and health-related quality of life [11,12]. In the present study, these functional assessments were included to provide complementary information on functional performance, whereas relative handgrip strength was selected a priori as the primary indicator of relative muscular function.

In adults with obesity, age-related neuromuscular decline may further impair muscular function, postural control, and movement efficiency, potentially worsening body composition and functional status [13]. Aging is associated with increased visceral adiposity and reduced muscle mass [14], and the concomitant presence of obesity and low muscle mass is commonly referred to as sarcopenic obesity [15]. Sarcopenia and obesity share several pathophysiological mechanisms, including hormonal alterations, physical inactivity, chronic inflammation, and elevated oxidative stress [16]. These mechanisms contribute to progressive declines in muscle quality, impaired neuromuscular function, and unfavorable changes in body composition, which may become more pronounced with advancing age. Consequently, the relationship between relative muscular function and body composition may differ across adulthood, particularly among women with obesity.

Furthermore, impairments in neuromuscular function appear to be strongly influenced by obesity severity in older adults [17].

Notably, individuals with obesity may exhibit greater absolute muscle strength values because excess body weight imposes continuous mechanical loads on the musculoskeletal system. However, when muscular strength is normalized to body mass, individuals with obesity generally show lower relative strength values compared with normal-weight individuals [17]. Relative handgrip strength, obtained by normalizing muscular strength to body weight, is increasingly recognized as a clinically relevant marker of muscular function and has been used to assess muscle function relative to body size [6,8]. Reduced relative strength may contribute to impaired postural stability, greater disability risk, and reduced efficiency during weight-bearing activities such as walking and stair climbing [18]. These limitations may also be associated with reduced participation in physical activity over time [19]. Unlike absolute handgrip strength, relative handgrip strength accounts for body weight and therefore provides a more meaningful estimate of muscle function in individuals with obesity.

Because excess body weight may artificially increase absolute strength values, normalizing strength to body weight offers a more accurate reflection of muscle quality and functional capacity, allowing better comparisons across individuals with different degrees of adiposity [7,8].

Previous studies in adults have shown that reduced relative handgrip strength is associated with higher adiposity, poorer body composition, reduced physical performance, and less favorable cardiometabolic profiles [7,17].

Moreover, relative handgrip strength progressively declines with aging and represents a robust marker of overall muscular fitness throughout adulthood [8].

However, most available studies have examined these relationships in heterogeneous adult populations without specifically investigating how these associations may differ across the aging process in women with obesity.

Therefore, the aim of the present study was to investigate age-related associations between relative handgrip strength, used as the primary indicator of relative muscular function, and body composition outcomes in women with obesity.

## 2. Materials and Methods

### 2.1. Participants

The present study is a secondary cross-sectional analysis of baseline data collected within a previously approved randomized controlled trial. Four hundred and nineteen female outpatients with obesity referred to the IRCCS Istituto Auxologico Italiano voluntarily participated in this research (age range 18–80 years, height 1.61 ± 0.12 m, body mass 102.7 ± 37.3 kg). Participants were consecutively recruited among women referred to the IRCCS Istituto Auxologico Italiano and enrolled in the institution’s standardized multidisciplinary weight-loss program between 1 October 2022 and 30 April 2024. All women meeting the eligibility criteria during the recruitment period were invited to participate in this secondary cross-sectional analysis based on baseline assessments performed before the start of the intervention. Only women were included because the number of men enrolled in the original randomized controlled trial was insufficient to allow meaningful sex-specific analyses. Furthermore, focusing on women was considered scientifically relevant, given the limited evidence specifically addressing the relationship between relative HST and body composition in women with obesity. The following inclusion criteria were observed: (i) an age between 18 and 80 years, (ii) a Body Mass Index of at least 30 kg/m^2^, according to the World Health Organization classification, and (iii) engaging in at least one structured physical activity session per week (minimum training duration: 60 min). Participants were stratified into age groups based on the sample distribution covering different stages of adulthood: 18–35 years (n = 38), 36–55 years (n = 141), and 56–80 years (n = 240). This age-group classification was adopted to examine whether the associations between relative handgrip strength and body composition differed across adulthood.

The exclusion criteria were presence of knee, hip, or spine pain or disability with a score greater than 7 out of 10 points on a visual analog scale, knee or foot osteoarthrosis, presence of neurological or psychiatric impairment, previous myocardial infarction, or any other medical disorder contraindicating participation in physical activity.

After a detailed explanation of the study’s aims, risks and benefits, participants signed a written informed consent form to participate. The research complies with the World Medical Association Declaration of Helsinki on studies with human participants and was approved by the institution ethics committee (approval number 2022_09_27_15).

### 2.2. Study Design and Setting

This cross-sectional study was conducted by analyzing baseline measurements in a single experimental session lasting approximately 40 min at IRCCS Istituto Auxologico Italiano under standardized environmental conditions (time of day 14:00–16:00, temperature 20–23 °C) before the start of a structured multidisciplinary, weight-loss lifestyle program [20,21,22].

### 2.3. Body Composition

Anthropometric outcomes were obtained by assessing body height in meters to the nearest 0.1 cm using a SECA 217 vertical stadiometer (SECA^®^ 240, Hamburg, Germany) and weight to the nearest 100 g using a SECA 877 scale (SECA^®^ 877, Hamburg, Germany). From these parameters, Body Mass Index (BMI) was calculated as bodyweight (kg) divided by height squared (m^2^). Regarding body composition, waist circumference (WC) was measured in standing position at the midpoint between the last rib and the iliac crest with a non-stretch tape to the nearest 0.5 cm.

Fat mass (FM%) and fat-free mass (FFM%) percentages were assessed by single-frequency bioelectric impedance analysis (BIA 101-RJL Systems Akern srl, Firenze, Italy). Measurements were performed with the patient in the supine position, with the lower limb abducted at 45 degrees from the midline section. Bioelectrical impedance analysis was performed after a 20 min rest in a ventilated, temperature-controlled room. Participants were instructed to fast overnight and not engage in physical activity in the previous 12 h. Resistance and reactance values were processed using BodygramPlus Enterprise software (version 1.2.9, Akern, Florence, Italy). According to the manufacturer’s documentation, fat mass and fat-free mass were estimated using the manufacturer’s proprietary regression algorithms. Bioelectrical impedance vector analysis (BIVA) was performed using the RXc graph based on raw resistance and reactance measurements.

Finally, waist-to-height ratio (WHtR) was calculated by dividing WC by height in centimeters and was considered an additional indicator of central obesity and cardiometabolic risk [23].

### 2.4. Physical Fitness

Functional performance was measured by adopting the modified version of the Functional Movement Screen protocol consisting of four out of seven movement patterns (i.e., deep squat, hurdle step, shoulder mobility, active straight leg raise) using a four-point graded scale (from 0 to 3 points) focused on the quality of movement pattern execution [24]. Each single score was summed to provide a final composite score (FMS CS).

Balance was assessed using the modified version of the Balance Error Scoring System (M-BESS) and was characterized by maintaining upright postural control in three positions for 20 s (i.e., bipodalic, tandem, and monopodalic stances). A higher inter-rater reliability was also established for this task (ICC = 0.88) [25]. In detail, the examiner counted the total number of errors an individual executed while maintaining the proper balance position, providing a proper composite score (M-BESS score).

Muscle strength was determined for both upper and lower limbs. Upper-limb strength was determined with the handgrip strength test using a Jamar hydraulic dynamometer (J. A. Preston Corporation, Clifton, NJ, USA) observing the standardized procedures [26]. Relative handgrip strength was calculated by normalizing handgrip strength values to body weight and was selected a priori as the primary indicator of relative muscular function and was therefore used as the independent variable in the regression analyses (relative HST). As for the lower limb, functional performance was assessed using the Five-Repetition Sit-to-Stand test (5STS). Participants were instructed to stand up and sit down five times as quickly as possible from a standardized chair without upper limb assistance. The total execution time was recorded in seconds [27].

Physical activity level was assessed using the International Physical Activity Questionnaire—Short Form (IPAQ-SF) [28]. The IPAQ-SF asks outpatients to report the amount of physical exercise performed during sessions lasting at least 10 min during the past week. Participants must declare the time dedicated to performing exercise at three intensities: walking pattern, moderate intensity, and vigorous intensity. Total weekly physical activity (MET min/week) was estimated by calculating the total time derived from each exercise type and intensity multiplied by its estimated metabolic equivalent of task (MET).

### 2.5. Statistical Analysis

Data are presented as mean ± standard deviation (SD). Data distribution was assessed using the Kolmogorov–Smirnov test [29]. Because variables were not normally distributed, non-parametric statistics were applied. Prior to model estimation, the assumptions of linear regression were assessed by visual inspection of standardized residual plots, histograms, and normal probability (P–P) plots. In the adjusted regression models, multicollinearity was evaluated using variance inflation factor (VIF) values, influential observations were assessed using Cook’s distance, and residual independence was evaluated using the Durbin–Watson statistic. Inspection of the regression diagnostics did not reveal major violations of linearity, homoscedasticity, normality of residuals, multicollinearity, influential observations, or residual independence. Participants were stratified into age groups based on the sample distribution, and differences across groups were assessed using the Kruskal–Wallis test.

Relative HST was selected a priori as the primary indicator of relative muscular function and was therefore entered as the independent variable in the regression analyses. The remaining functional assessments (FMS, M-BESS, and 5STS) were analyzed descriptively to characterize functional differences across age groups.

Separate linear regression analyses were performed within each age group to investigate the associations between relative HST (independent variable) and body composition outcomes, including FM%, FFM%, WC, and WHtR. To formally assess whether these associations differed across age groups, additional pooled linear regression models included age group, relative HST, and their interaction term (relative HST × age group), with IPAQ-SF entered as a covariate. The overall interaction was evaluated using a joint F test of the two interaction coefficients, with the 18–35-year group as the reference category. BMI was not included as a covariate because relative HST was calculated by normalizing handgrip strength to body weight, while body weight is also a component of BMI. Including BMI in the same model could therefore introduce mathematical coupling and complicate the interpretation of the independent association between relative HST and body composition outcomes.

In addition, linear regression models adjusted for age and physical activity level (IPAQ-SF) were employed in the whole sample to determine whether the associations between relative HST and body composition outcomes remained independent of these potential confounders. A post hoc sensitivity analysis was conducted using G*Power 3.1 software (α = 0.05, power = 0.80) to estimate the minimum detectable effect size for each predefined age group. The analysis yielded minimum detectable Cohen’s f^2^ values of 0.218 (n = 38), 0.056 (n = 141), and 0.033 (n = 240), indicating progressively greater sensitivity to detect smaller associations with increasing sample size.

Statistical significance was set at *p* < 0.05. The original analyses were performed using IBM SPSS Statistics version 21.0. The additional pooled interaction analyses were performed using Python 3 with statsmodels version 0.14.6. As this study represents a secondary analysis of an existing dataset, no additional a priori sample size calculation was performed specifically for the present analysis.

### 2.6. Use of AI-Assisted Tools

The authors used an AI-assisted language tool (ChatGPT 5.5, Open AI, San Francisco, CA, USA) in keeping with established best practices [30] to edit wording and improve readability. This tool was not involved in the generation of data, results, or interpretations. The final content has been thoroughly revised, edited, and approved by the authors.

## 3. Results

All 419 women were included in the descriptive analyses. A small number of values were unavailable for selected variables; therefore, regression models were estimated using complete cases for the variables included in each model, and the effective sample size varied slightly across outcomes. Participants were subsequently stratified into age groups according to sample distribution. Descriptive anthropometric, body composition, and physical activity characteristics according to age groups are presented in Table 1. Significant differences across groups were observed for BMI and FM%, whereas no significant differences emerged for WC, FFM%, WHtR, or physical activity level assessed by IPAQ-SF.

**Figure 1:**
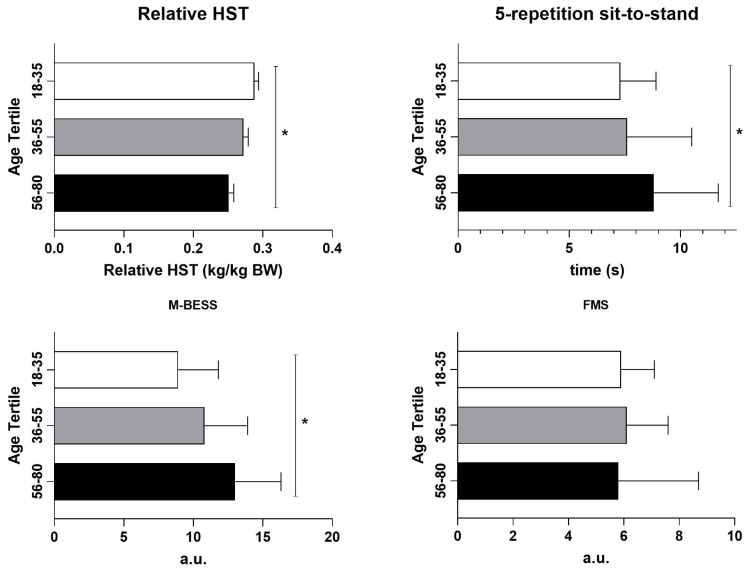
Age-related differences in motor function outcomes across age groups. Data are presented as mean ± standard deviation (SD). Older participants showed lower relative handgrip strength, poorer balance performance (M-BESS), and reduced lower-body functional performance (5STS), whereas no significant differences were observed for FMS CS score. * Statistically significant differences among age groups (*p* < 0.05).

Age-related differences in motor function outcomes are presented in Figure 1. Relative HST (*p* = 0.0002), balance performance assessed through the M-BESS (*p* < 0.0001), and 5STS performance (*p* < 0.0001) differed significantly across groups, whereas no significant differences were observed for FMS CS score (*p* = 0.2301). Older participants showed lower relative HST together with poorer balance and lower functional performance.

Linear regression analyses according to age groups were performed using relative HST as the independent variable to examine its associations with body composition outcomes. Table 2 presents standardized (β) and unstandardized (B) regression coefficients with their corresponding 95% confidence intervals (95% CI), *p* values, and adjusted R^2^ for each model. In the youngest age group (18–35 years), relative HST was significantly associated with lower FM% (β = −0.630, *p* < 0.001) and higher fat-free mass (FFM%) values. Conversely, no significant associations were identified for WC or WHtR outcomes, although the corresponding regression coefficients and 95% confidence intervals are reported in Table 2 for completeness.

In the middle-aged women (36–55 years), relative HST was independently associated with lower FM%, higher FFM%, and lower WC values. No significant associations emerged for WHtR.

Similarly, in the oldest age group (≥56 years), higher relative HST was associated with lower FM%, higher FFM%, and lower WC. No significant associations were found between relative HST and WHtR across age groups.

To formally assess whether the age-stratified associations differed across age groups, pooled linear regression models including a relative HST × age-group interaction term were additionally performed. No significant interaction was observed for FM% (*p* = 0.680), FFM% (*p* = 0.330), waist circumference (*p* = 0.838), or WHtR (*p* = 0.869), indicating that age group did not significantly modify these associations.

Additional regression analyses performed in the whole sample adjusting for age and physical activity level (IPAQ-SF) showed that the model explained 28.9% of the variance in FM% (R^2^ = 0.290, *p* < 0.001), with relative HST (β = −0.529, *p* < 0.001) and age (β = −0.226, *p* < 0.001) emerging as significant predictors, whereas physical activity was not independently associated with FM% (*p* = 0.501). For WC, the model explained 3.3% of the variance (R^2^ = 0.033, *p* = 0.001), with both relative HST (β = −0.171, *p* = 0.001) and age (β = −0.101, *p* = 0.043) remaining significantly associated with WC, while no significant association was observed for physical activity (*p* = 0.809). In contrast, in the fully adjusted whole-sample models, no independent association between relative HST and FFM%, WHtR, or BMI was observed after adjustment for age and physical activity.

Thus, although the age-stratified analyses showed some descriptive differences in the pattern of significant associations, the estimated relative HST–body composition relationships did not differ significantly across age groups.

## 4. Discussion

The present study investigated age-related associations between relative HST and body composition in women with obesity.

The present study provides three main findings. First, relative HST was significantly associated with body composition in younger women with obesity, particularly with FM% and FFM%.

Second, WC was significantly associated with relative HST in middle-aged women, in addition to FM% and FFM%, whereas the corresponding WC association was not statistically significant in the youngest group. However, the formal interaction analysis did not indicate that the WC–relative HST association differed significantly across age groups. The WC model within the middle-aged group also explained only a small proportion of the outcome variance (adjusted R^2^ = 0.028), indicating limited explanatory power despite statistical significance. Therefore, these findings should be interpreted cautiously, as additional biological and lifestyle factors are likely to contribute to waist circumference in this population.

Nevertheless, these findings may indicate a pattern of associations between relative HST and body composition during middle adulthood, including central adiposity.

The association observed within middle-aged women may partly reflect factors related to the menopausal transition, during which estrogen decline promotes visceral fat redistribution and reductions in muscle mass and strength [31,32]. Nevertheless, because the interaction test was not significant and menopausal status was not directly assessed, this interpretation remains speculative. Associations with FM% and FFM% were also maintained in older women, supporting the relevance of relative HST as a marker of muscle-related functional status across adulthood.

Third, although the age-stratified analyses suggested variation in the pattern of associations between relative HST and body composition across adulthood, the formal pooled interaction analyses indicated that age group did not significantly modify these associations. Therefore, the observed differences between age groups should be interpreted descriptively rather than as evidence of true effect modification.

In contrast, FMS composite score did not differ significantly across age groups. This finding suggests that movement quality, as assessed by the Functional Movement Screen, may be less sensitive to age-related differences than relative HST, balance performance, or lower-body functional performance in women with obesity. It is also possible that FMS primarily reflects movement quality and mobility rather than the age-related neuromuscular decline captured by relative handgrip strength and the other functional assessments.

Taken together, these findings reinforce the relevance of relative HST in the context of obesity and age-related functional decline. Rather than demonstrating a previously unreported association between relative HST and body composition, the novelty of the present study lies in examining these associations across different stages of adulthood in women with obesity and formally testing whether age group modified them, a topic that has been comparatively understudied.

Although previous evidence has consistently shown inverse associations between adiposity and motor competence in pediatric populations [10,24,33,34], these studies primarily provide developmental context and should not be directly extrapolated to adults with obesity because the determinants of motor competence and relative muscular function differ substantially across the lifespan.

However, translating these findings to adulthood is not straightforward because adults with obesity typically present long-standing alterations in body mass distribution, musculoskeletal loading, and neuromuscular control, which may further impair movement efficiency and postural stability [35].

Aging is associated with progressive alterations in body composition and neuromuscular function, including increased visceral adiposity and reduced muscle mass [14]. The concomitant presence of obesity and low muscle mass, commonly defined as sarcopenic obesity [15], is strongly associated with poorer physical function, disability risk, and reduced quality of life. Sarcopenia and obesity share several pathophysiological mechanisms, including hormonal alterations, chronic inflammation, low physical activity levels, and oxidative stress [16].

The age-stratified analyses showed some descriptive variation in the pattern of associations between relative HST and body composition. WC was significantly associated with relative HST in the middle-aged and older groups, whereas FM% and FFM% were associated with relative HST across all age groups, with the largest standardized coefficients for these variables observed in the youngest group. These descriptive findings are consistent with the pooled interaction analyses, indicating that the apparent differences across age groups should not be interpreted as evidence of true effect modification.

In contrast, no significant associations were observed between relative HST and WHtR in any age group. Although WHtR is widely considered a robust indicator of central adiposity and cardiometabolic risk, our findings suggest that WHtR and WC may capture different aspects of central adiposity in relation to muscular function. The lack of association with WHtR indicates that its value as an indicator of cardiometabolic risk does not necessarily translate into a closer relationship with relative muscular function in women with obesity [36].

Furthermore, aging was accompanied by lower relative HST, poorer balance performance, and reduced lower-body functional capacity, supporting the role of muscular function as a clinically relevant marker of sarcopenic obesity and functional decline. Notably, additional regression analyses performed in the whole sample after adjustment for both age and physical activity level (IPAQ-SF) confirmed that the associations between relative HST and both FM% and waist circumference remained significant, suggesting that these relationships were not explained by either age or habitual physical activity. Moreover, physical activity was not independently associated with any of the investigated body composition outcomes.

Consistent with the previous literature, fat-free mass appeared to be more strongly associated with muscular function than traditional anthropometric indicators such as BMI or waist circumference [6,32]. Relative HST was significantly associated with fat-free mass parameters across all age groups, supporting its role as a functional marker of muscle quality independently from body size. Individuals with greater muscular function may also exhibit more favorable body composition profiles [37,38,39]. However, the present study was not designed to investigate the biological mechanisms underlying these associations, and therefore, mechanistic interpretations should be considered with caution.

From a clinical perspective, the assessment of relative HST may provide complementary information to traditional anthropometric measures when evaluating functional status in individuals with obesity. Excess adiposity alters body geometry and shifts the center of mass, increasing postural and balance demands during daily activities [40]. Therefore, monitoring outcomes such as relative handgrip strength, balance performance, and lower-body functional capacity may help identify clinically relevant functional limitations.

These findings reinforce the importance of integrating functional assessment into multidisciplinary obesity management. The evaluation of relative HST may support the development of more individualized and feasible exercise interventions aimed at improving functional autonomy, physical activity participation, and quality of life. Although several functional outcomes were assessed, relative HST was selected a priori as the primary indicator because it represents a simple, clinically feasible and valid indicator of relative HST in women with obesity.

Nevertheless, the results of the present study should be interpreted considering some limitations. First, the cross-sectional design does not allow causal relationships to be established, and reverse causation cannot be excluded, as poorer body composition may also contribute to lower relative muscular function. Age was categorized into groups to facilitate clinically interpretable comparisons and interaction testing. Nevertheless, categorization may reduce statistical information and power; modeling age continuously, for example, using flexible nonlinear terms, may provide additional information in future studies. Second, the study included only female participants, limiting the generalizability of the findings to males, who may present different relative strength values and body composition characteristics. In addition, information regarding pharmacological treatments and relevant comorbidities potentially affecting muscular function was not systematically collected and therefore could not be considered in the analysis. Third, participants were required to perform at least one structured physical activity session per week (minimum duration: 60 min) as an eligibility criterion. Consequently, the present findings may not be fully generalizable to completely sedentary women with obesity. Furthermore, participants were recruited from a specialized obesity center and enrolled in a standardized multidisciplinary weight-loss program. Therefore, the findings may not be fully generalizable to women with obesity managed in primary care or other community-based clinical settings. The recruitment of participants from a specialized clinical cohort may also have introduced selection-related bias, which should be considered when interpreting the observed associations.

Fourth, relative HST was assessed by normalizing handgrip strength to body weight, without a direct imaging-based assessment of muscle quality.

Because relative HST is derived by normalizing handgrip strength to body weight, some degree of mathematical coupling with body weight-related outcomes (e.g., BMI, waist circumference, waist-to-height ratio, and body composition measures) cannot be excluded and should be considered when interpreting the observed associations. Finally, body composition was evaluated using bioelectrical impedance analysis. Although BIA is widely used in clinical practice [41], the estimation of fat mass and fat-free mass may be influenced by hydration status and tissue characteristics when compared with dual-energy X-ray absorptiometry. Moreover, because the present cohort was characterized by a high mean BMI, the accuracy of BIA may be reduced in individuals with severe obesity, potentially affecting the estimation of fat mass and fat-free mass and, consequently, influencing the magnitude of the observed associations [42]. Although physical activity level was included as a covariate in the adjusted regression analyses and did not differ significantly across age groups, it was assessed using the self-reported IPAQ-SF questionnaire. Therefore, residual confounding related to habitual physical activity cannot be completely excluded, and the findings should be interpreted with appropriate caution.

## 5. Conclusions

Relative HST was consistently associated with body composition outcomes across women with obesity. Although the age-stratified models showed descriptive differences in which outcomes reached statistical significance, the pooled interaction analyses did not provide evidence that age group significantly modified these associations.

The observed associations support the potential clinical relevance of relative HST as a marker of muscular fitness in women with obesity. Its assessment may provide complementary information during the functional evaluation of women with obesity.

However, longitudinal studies are needed to determine whether relative HST has additional utility for individualized and patient-centered obesity management, and to clarify the temporal relationships between relative HST, body composition, and age-related changes in women with obesity.

## Figures and Tables

**Table 1:** Age group stratification and descriptive analysis.

Variable	Age Group 1 (18–35 Years, n = 38)	Age Group 2 (36–55 Years, (n = 141)	Age Group 3 (56–80 Years, n = 240)	*p* Value
Age (y)	26.6 ± 4.5	49.2 ± 4.9	63.2 ± 5.4	<0.0001
BMI (kg/m^2^)	40.3 ± 6.4	41.7 ± 3.1	37.2 ± 5.4	0.0006
WC (cm)	120.6 ± 16	119.4 ± 13.6	118.2 ± 10.8	0.8959
FM (%)	50.5 ± 5.2	49 ± 6	48 ± 5.7	0.0308
FFM (%)	49.7 ± 5.3	51.1 ± 5.9	51.8 ± 5.7	0.0798
WHtR (WC cm/height cm)	0.7 ± 0.1	0.7 ± 0.1	0.8 ± 0.07	0.5036
IPAQ-SF (MET min/week)	827.4 ± 2847.0	353.2 ± 334.0	368.6 ± 701.0	0.659

Note: y = years; BMI = Body Mass Index; WC = waist circumference; FM = fat mass; FFM = fat-free mass; WHtR = waist-to-height ratio; IPAQ-SF = International Physical Activity Questionnaire—Short Form.

**Table 2:** Linear regression analysis between relative handgrip strength and body composition outcomes across age groups.

Age Groups	Outcome	Predictor	β	B (95% CI)	*p* Value	Adjusted R^2^
18–35	FM%	Relative HST	−0.630	−51.92 (−73.54 to −30.31)	<0.001	0.381
18–35	FFM%	Relative HST	0.583	48.66 (25.73 to 71.59)	0.001	0.321
18–35	WC	Relative HST	−0.133	−33.42 (−117.58 to 50.73)	0.426	−0.010
18–35	WHtR	Relative HST	−0.088	0.12 (−0.58 to 0.34)	0.598	−0.020
36–55	FM%	Relative HST	−0.537	−48.07 (−60.79 to −35.36)	<0.001	0.283
36–55	FFM%	Relative HST	0.543	47.10 (34.84 to 59.36)	<0.001	0.290
36–55	WC	Relative HST	−0.188	−38.07 (−71.62 to −4.52)	0.026	0.028
36–55	WHtR	Relative HST	−0.028	−0.05 (−0.33 to 0.24)	0.740	−0.006
56–80	FM%	Relative HST	−0.504	−42.17 (−51.40 to −32.93)	<0.001	0.251
56–80	FFM%	Relative HST	0.423	35.88 (26.08 to 45.68)	<0.001	0.176
56–80	WC	Relative HST	−0.166	−26.46 (−46.55 to −6.36)	0.010	0.024
56–80	WHtR	Relative HST	−0.052	−0.51 (−1.77 to 0.75)	0.423	−0.001

Note: WC = waist circumference; FM% = fat mass; FFM% = fat-free mass; WHtR = waist-to-height ratio; Relative HST = relative handgrip strength test normalized to body weight; β = standardized regression coefficient; B = unstandardized regression coefficient; CI = confidence interval.

## Data Availability

The data presented in this study are available on request from the corresponding author. The data are not publicly available due to privacy reasons.
